# Optimized process for fabrication of free-standing silicon nanophotonic devices

**DOI:** 10.1116/1.4983173

**Published:** 2017-05-12

**Authors:** Paul Seidler

**Affiliations:** IBM - Research – Zurich, Säumerstrasse 4, CH-8803 Rüschlikon, Switzerland

## Abstract

A detailed procedure is presented for fabrication of free-standing silicon photonic devices that accurately reproduces design dimensions while minimizing surface roughness. By reducing charging effects during inductively coupled-plasma reactive ion etching, undercutting in small, high-aspect ratio openings is reduced. Slot structures with a width as small as 40 nm and an aspect ratio of 5.5:1 can be produced with a nearly straight, vertical sidewall profile. Subsequent removal of an underlying sacrificial silicon dioxide layer by wet-etching to create free-standing devices is performed under conditions which suppress attack of the silicon. Slotted one-dimensional photonic crystal cavities are used as sensitive test structures to demonstrate that performance specifications can be reached without iteratively adapting design dimensions; optical resonance frequencies are within 1% of the simulated values and quality factors on the order of 10^5^ are routinely attained.

## I. INTRODUCTION

Process technology for the fabrication of silicon photonic devices is already well established, the development of which has leveraged the extensive previous knowledge in the field of microelectronics.^1^ Nevertheless, the dimensional and geometric specifications of the most aggressively miniaturized photonic devices, such as microresonators,^2^ photonic crystals,^3^ and optoelectromechanical devices,^4,5^ still present challenges. Some of the requirements for achieving the desired performance targets, such as resonance frequency and quality factor (Q), of these so-called nanophotonic devices are dimensional accuracy (on the order of a few nanometers), sidewall angle (typically vertical within a few degrees), and sidewall roughness (on the order of 1/1000 of the vacuum wavelength). In addition, many structures are required to be free-standing, which puts demands on both material and process compatibilities. Often an iterative procedure is required, in which the design geometry is adjusted in a series of repeated fabrication cycles to compensate for the distortions occurring during processing.^6^

Dry etching methods using various halide-based (e.g., SF_6_, Cl_2_, or HBr)^7–9^ chemistries provide the standard processes for lithographic pattern transfer into silicon. Especially for devices where part or all of the structure has dimensions well below 100 nm, competing requirements on process conditions and behavior, such as the formation of passivation layers, often arise. This can result in unacceptable profiles including, for example, markedly sloped sidewalls or undercutting.^10,11^

Free-standing devices are typically created by removal of an underlying sacrificial silicon dioxide layer using wet etching with hydrofluoric acid (HF) chemistry, either concentrated HF (≥48%) or standard buffered oxide etch.^12–14^ As comparatively large amounts (1 *μ*m or more) of oxide must be removed, high selectivity with respect to silicon must be ensured. If the silicon is even slightly attacked, the device performance can be unacceptably compromised.

This paper describes a particular method for fabrication of free-standing silicon photonic devices that faithfully reproduces design dimensions while achieving the necessary surface roughness and cross-sectional geometry of the desired structures. Specifically, an approach is presented to avoid a bowed or undercut profile during inductively coupled-plasma reactive ion etching (ICP-RIE) with HBr/O_2_ chemistry of small, high-aspect ratio openings, which at the same time maintains nearly vertical sidewalls. The subsequent wet-etching procedure for releasing free-standing devices under conditions which suppress attack and concomitant roughening of the silicon surface is explained in detail. The benefits are demonstrated with an example of the performance of a slotted one-dimensional (1D) photonic crystal nanobeam cavity, a design which is especially sensitive to dimensional accuracy.^15,16^

## II. MODELING

While the necessity of an exact and controlled method of fabrication holds for numerous device designs, we illustrate here the constraints on the process technology by examining first one specific device structure. Using a freely available software package^17^ for finite-difference time-domain (FDTD)^18^ simulations, we investigate the tolerance to variations in certain dimensions.

**Fig. 1.**
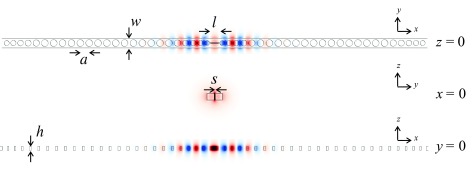
(Color) Schematic drawing of the optimized photonic crystal nanobeam cavity with a=470 nm, w=510 nm, h=220 nm, s=40 nm, and l=564 nm. The hole radius is given by r=0.370a. The five holes on either side of the central slot as well as the five holes at each end of the device are linearly tapered in spacing and radius to 72% of their nominal value. The color scale indicates the amplitude (blue and red corresponding to opposite signs) of the $E_{y}$ component of the electric field of the first even mode for two-dimensional cross-sections through the center of the cavity taken from a FDTD simulation.

A schematic of the optimized geometry is shown in Fig. 1. The 1D photonic crystal cavity comprises a freestanding waveguide with a sequence of holes forming Bragg mirrors on either side of a central slot. The holes have a nominal period of a=470 nm, with device width w=510 nm, device height h=220 nm, slot width s=40 nm, and slot length l=564 nm. While the periodic hole radius is given by r=0.370a=174 nm, the five holes closest to the slot as well as the five holes farthest from the slot on each side of the structure are linearly tapered in both radius and spacing to 72% of their nominal value for purposes of impedance matching.^19,20^ In other words, the tapered regions gradually match the effective evanescent mirror Bloch mode index of the periodic holes to the effective index of a simple straight waveguide at both the center and ends of the device. FDTD simulations of this structure using an index of refraction for silicon of $n_{\text{Si}}=3.48$ predict the presence of an even mode (symmetric with respect to both the x=0 and y=0 mirror planes) at $\lambda_{vac}=1554\;\text{nm}$ with a mode volume of 0.011 ${\left(\lambda_{vac}/n\right)}^{3}$, where n is the index of refraction at the location of maximum electric field magnitude (in this case in the slot, so n=1) and $\lambda_{vac}$ is the vacuum wavelength of the light.

The performance of the device can be characterized by its quality factor Q, which is defined by $Q=2\pi \nu_{0}\left(U/P\right)$, where $\nu_{0}$ is the resonance frequency, U is the electromagnetic energy in the cavity, and P is the rate of energy loss, in other words, the power leaving the cavity. Here, the simulated value of Q is 1.7 × 10^6^. Experimentally, Q can be determined by fitting the observed transmission peak with a Lorentzian function and using the relation $Q=\nu_{0}/\Delta \nu$, where Δν is the full width at half maximum.

**Fig. 2.**
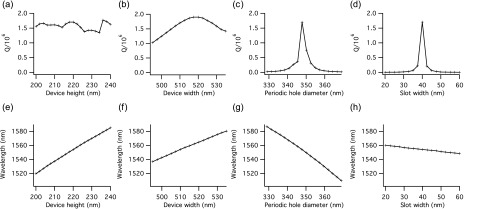
Dependence of (a)–(d) Q and (e)–(h) resonance wavelength on device dimensions as determined by FDTD simulations. In each case, only the dimension indicated was varied while all other dimensions remain as given in Fig. 1.

Figure 2 shows the quality factor Q and the resonance wavelength of the device as a function of four dimensions which could be influenced by fabrication conditions and are thus process dependent. (In each case, only the indicated dimension is varied while all others are held fixed.) Other dimensions, such as the slot length l and the position of the hole centers, are essentially determined by the accuracy of the e-beam writing used to define them (see below) and are therefore independent of subsequent process steps. It is also to be expected that, in practice, the height h of the device will vary less than the other dimensions, such as the width w, because the top silicon thickness is in principle fixed by the choice of starting wafer, whereas the lateral dimensions are more strongly dependent on process conditions. This is not to say however that the silicon thickness is immutable.

The simulations clearly indicate that the Q is only moderately affected by the width and height of the structure. A deviation of even 20 nm in either dimension can be tolerated, as the Q remains on the order of 10^6^. In contrast, the nominal diameter of the periodic holes and, especially, the slot width have a marked influence on the Q of the device; 1–2 orders of magnitude in Q are lost with a deviation of just a few nanometers. Interestingly, the slot width has the least influence on the resonance wavelength. Accurate reproduction of the design values for width, height, and hole diameter is more essential to achieving the desired resonance wavelength, with less silicon in general corresponding to a blueshift, as would be expected for a reduction in the proportion of high-index material present.^3^ Overall, it is evident that a fabrication accuracy of 5 nm or better is required, if values close to the target Q and resonance wavelength are to be achieved. In practice, deviations from the design dimensions may occur not only in the lateral outline of the structure, but also in the sidewall profile. Having the correct profile in the narrow central slot is particularly important.

## III. EXPERIMENTAL RESULTS

Nanobeam photonic crystal cavities of various designs were fabricated on 2 × 2-cm chips diced from a ⟨100⟩-oriented silicon-on-insulator (SOI) wafer with a nominal top silicon thickness of 220 nm and either a 2 or 3-*μ*m buried oxide layer. The layout included input and output ridge waveguides with a length of several hundred microns connected to each device. Focusing grating couplers at the ends of the waveguides were used for transmission measurements.^15,21^ The photonic crystal cavities, waveguides and grating couplers were patterned together in the top silicon by e-beam lithography and dry etching. The device portion of the structures was made free-standing using photolithography and wet etching.

### A. Dry etching of silicon

Device structures were defined by means of e-beam lithography using a Vistec EBPG 5200ES system and 4% hydrogen silsesquioxane (HSQ) in 4-methylpentan-2-one (methylisobutyl ketone) from Dow Corning as negative resist. Proximity-error-corrected layouts were written with a 5-nm bias, i.e., 5 nm were added with respect to the design geometry in the direction normal to the outline of the silicon. Prior to spin coating with HSQ, substrate chips were cleaned with a hot Piranha solution consisting of a 1:1 mixture of concentrated H_2_SO_4_ (96%) and H_2_O_2_ (30%–31%) for 5 min followed by a deionized-water rinse also for 5 min. A two-step spin-coating process was employed, namely 5 s at 500 rpm followed by 60 s at 6000 rpm (4000 rpm/s ramps), resulting in a HSQ film thickness of typically 85–95 nm.

Pattern transfer into the top silicon was accomplished by inductively coupled-plasma reactive ion etching (Oxford Plasmalab System 100 ICP) with HBr/O_2_ chemistry. The samples were affixed to a 100-mm silicon carrier wafer with a dab of medium-temperature hydrocarbon vacuum grease (Apiezon^®^ T, M&I Materials), which could be easily removed after etching with a nonpolar solvent such as hexane. The carrier wafer was mechanically clamped by a quartz ring. Whereas there are a range of process conditions suitable for etching of the desired silicon structures, we typically used one of two recipes with the parameters shown in Table I. In both cases, the chamber pressure was 4.0 mTorr, and the lower electrode temperature was 50 °C with backside helium cooling of the carrier wafer. The HBr-only step is intended to break through any native oxide layer covering the silicon. The presence of O_2_ in the second step serves to passivate and protect the side walls of the silicon structure as well as enhance the Si/SiO_2_ selectivity. Hence, the O_2_ content can be used together with the bottom electrode temperature to tune the side wall angle,^8,9,22^ and, in general, a higher O_2_-to-HBr ratio is required at higher ICP power for a given sidewall angle. The HSQ mask was removed after dry etching by submerging the chip in standard buffered oxide etchant (BOE), a 7:1 mixture by volume of 40% NH_4_F in water to 49% HF in water (Technic), for 10 s.

**Table I.** Parameters for dry etching of silicon. The overall etch rate for recipe I is 2.4–2.6 nm/s with a selectivity of 7.5:1 for silicon with respect to SiO_2_. For recipe II, these values are 1.7–1.8 nm/s and 8.9:1, respectively.

Recipe	Step	Duration (s)	HBr flow (sccm)	O_2_ flow (sccm)	RF power (W)	ICP power (W)
I	1	10	40	—	110	800
	2	90	37	3.0	110	600
II	1	10	40	—	100	600
	2	130	39	1.0	100	250

**Fig. 3.**
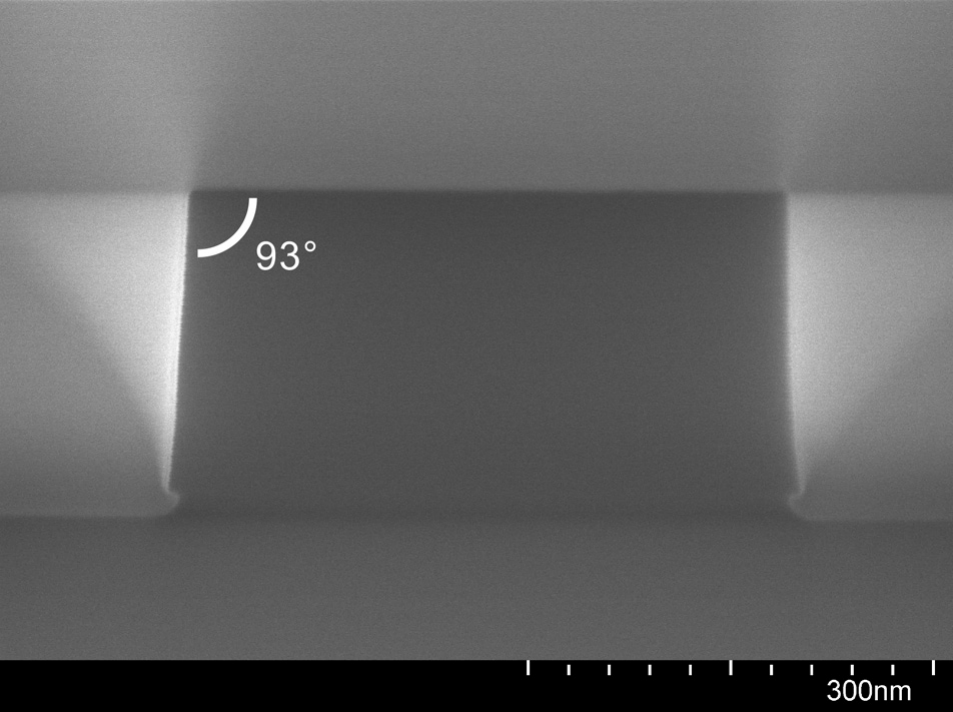
SEM image at 180 000× magnification of a cleaved chip showing the cross-section of a silicon waveguide following dry etching with recipe II and removal of the HSQ mask with BOE.

Recipes I and II were chosen as a compromise to give nearly vertical outer sidewalls (see Fig. 3) of the nanobeam photonic crystal cavities while at the same time permitting the formation of small, high-aspect-ratio slot structures. The latter require additional passivation to avoid undercutting and formation of a bowed profile. Indeed, it is well known that etching of such features presents at least two, sometimes competing, challenges: (1) transport of etchant species in and reaction products out of small openings and (2) increased influence of charging by ions and electrons, particularly with insulating materials.^10,11^ For the structures investigated here, the predominant issue appears to be charging, the effects of which become pronounced for openings with lateral dimensions at or below ca. 60 nm. The observed sidewall profile is attributed to the less well confined electrons in the ICP-RIE chamber leading to negative charging of the poorly conducting HSQ layer on top of the chip and, consequently, to deflection toward the sidewalls near the mouths of small openings of the positive ions impinging on the sample. The result is often a keyhole-shaped vertical profile.

**Fig. 4.**
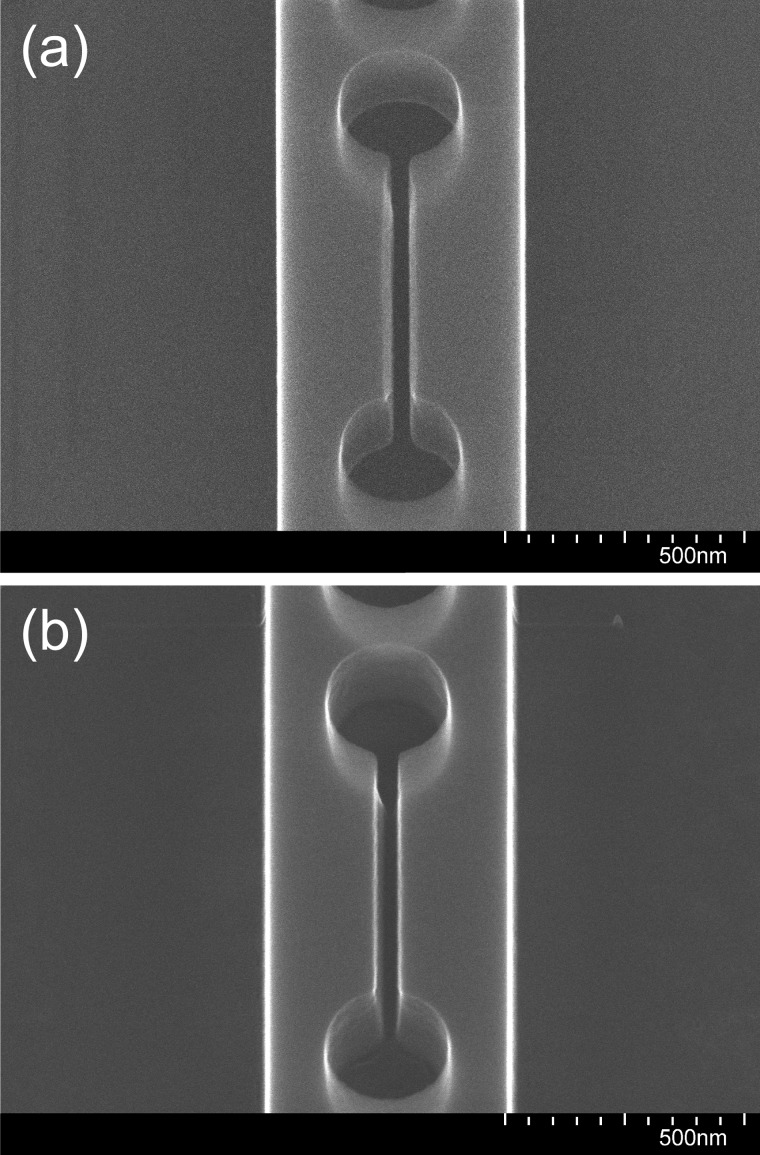
SEM images at 80 000× magnification and 30° tilt angle of the central slot of a slotted photonic crystal nanobeam cavity from two different chips following dry etching and removal of the HSQ mask with BOE.

Two examples of the sort of slot profile that can be produced depending on the etching parameters are shown in Fig. 4 for a nominally 40-nm wide slot. For the sample shown in Fig. 4(a), the HBr and O_2_ flow rates in the second etch step were 37.5 and 2.5 sccm, respectively, and the RF power was 90 W. The top of the slot has been undercut, but the bottom of the slot appears to be nearly vertical. In contrast, Fig. 4(b) displays a sample for which the parameters of step 2 were as in recipe I. Here the slot is more V-shaped, indicating that the higher O_2_ fraction perhaps leads to too much passivation of the sidewalls.

The erosion of the sidewalls is often asymmetric, that is, the side of the slot facing the center of the chip is etched more, an observation confirmed by examining devices on opposite sides of the chip. [In Figs. 4(a) and 4(b), the center of the chip is to the right of the device shown.] This is consistent with an overall buildup of charge on the surface of the chip, leading to steering of the etchant species toward the edges.

**Fig. 5.**
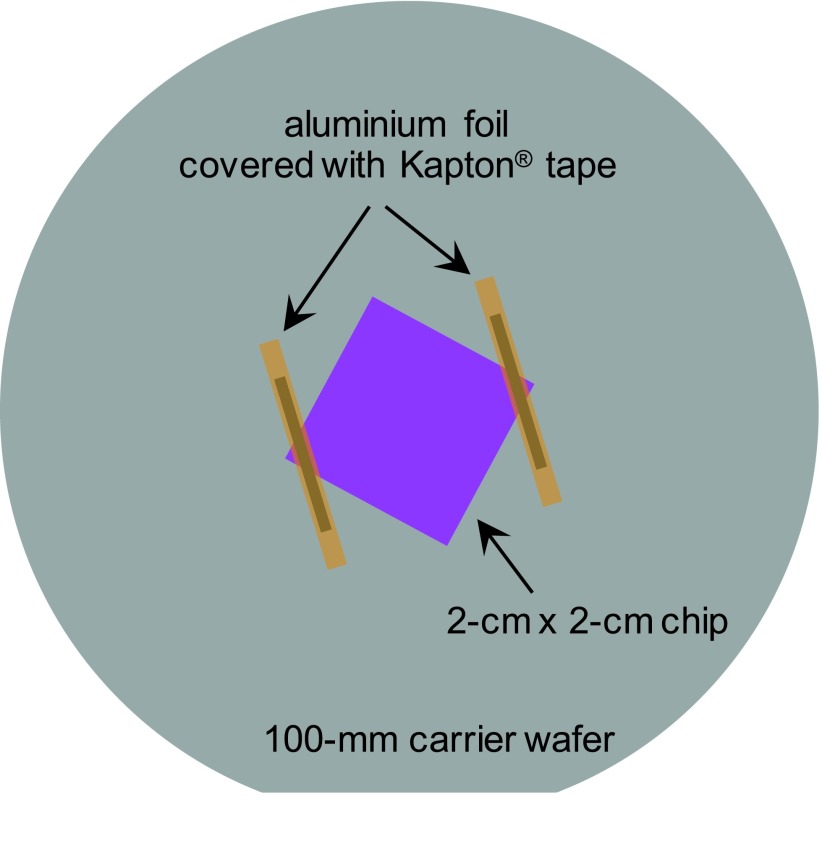
(Color) Schematic of sample chip mounted on a carrier wafer with strips of aluminum foil covered with Kapton tape.

**Fig. 6.**
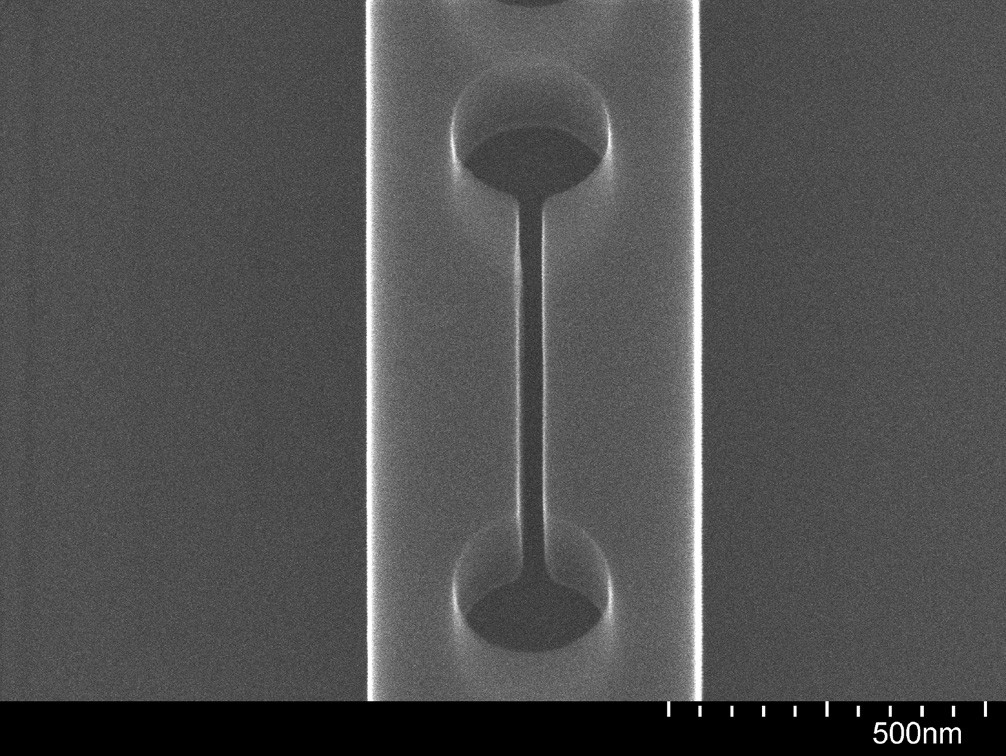
SEM image at 80 000× magnification and 30° tilt angle of the central slot of a slotted photonic crystal nanobeam cavity following dry etching and removal of the HSQ mask with BOE, where aluminum strips connecting the top surface of the chip to the carrier wafer were used.

To address this issue, an electrically conductive path was made between the top of the sample and the carrier wafer by placing strips of aluminum foil over two opposite corners of the chip and covering them with polyimide tape (Kapton^®^) to fix them in place and prevent their exposure to the etching environment, as illustrated in Fig. 5. The benefits of the electrical connection are expected to last as long as some of the top-silicon layer is still present. It is therefore important to limit the etch time so as not to over-etch for an extended period. The advantage of this procedure is evident from Fig. 6, where the sidewalls of a 40-nm slot are seen to be nearly straight and vertical. The aspect ratio is 5.5:1.

### B. Device release

**Fig. 7.**
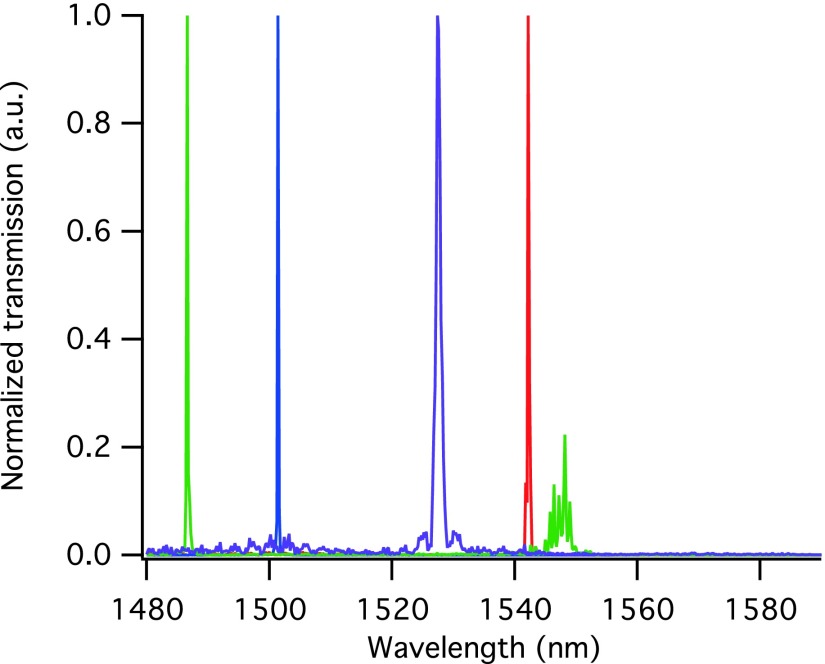
(Color) Normalized transmission spectra of slotted photonic crystal nanobeam cavities with ostensibly the same design resonance frequency. In the case of the green spectrum, the resonance of interest is so strongly blue-shifted, that a second, broader resonance becomes visible in the observed wavelength range.

In order to create freestanding devices, the SiO_2_ underlying the top silicon layer is removed by a procedure involving photolithography and wet etching. The obvious approach would be to first coat the sample with a typical positive resist such as AZ^®^ 6612 (AZ Electronic Materials) and expose it with a mask defining openings around the photonic crystal cavities and leaving any connecting structures such as waveguides and grating couplers protected. The sacrificial buried-oxide layer under the devices would then be removed using standard BOE. With this etchant, 15 min are required for the removal of approximately 1.1 *μ*m of SiO_2_. Unfortunately, we find that this straightforward recipe yields devices of variable optical performance, as determined by optical quality factor, and with resonance frequencies that are always blue-shifted with respect to the simulated design values. Indeed, there can be considerable variation on a single chip (see Fig. 7). The higher resonance frequency (shorter wavelength) suggests that overall less silicon is present in the device structure than in the design geometry, although which dimensions are inaccurate is not obvious from the optical transmission measurements.

**Fig. 8.**
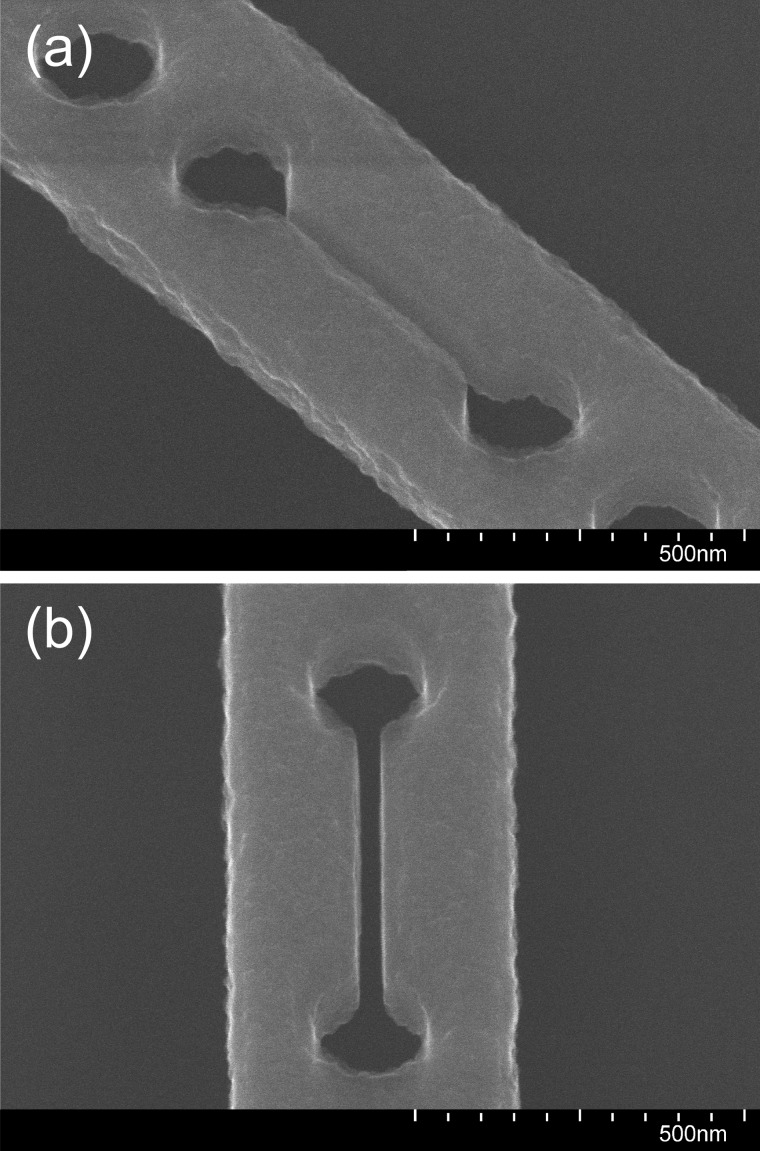
SEM images at 110 000× magnification and 30° tilt angle of the central slot region of the same fully processed, free-standing, slotted photonic crystal nanobeam cavity viewed at two different angles.

**Fig. 9.**
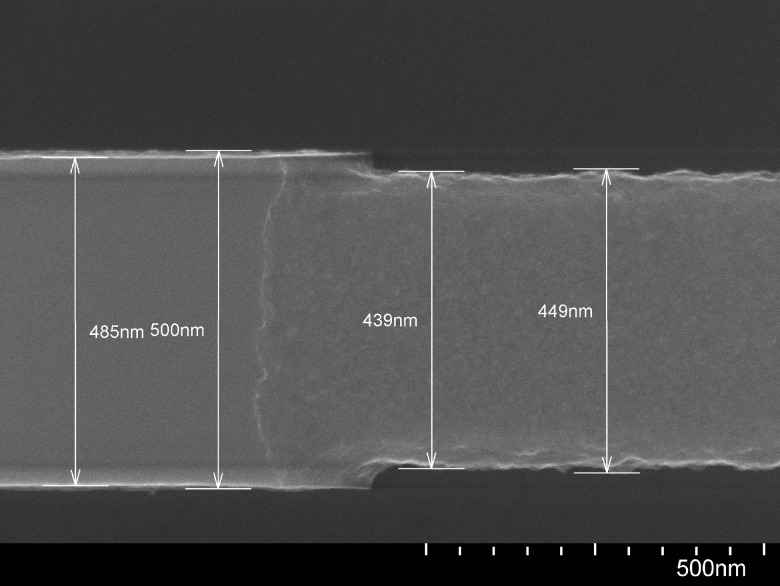
SEM image at 110 000× magnification of the waveguide at the edge of the wet-etched opening where the device becomes free-standing.

Examination with a scanning electron microscope (SEM) of a device where the blueshift was particularly pronounced (Fig. 8) revealed the origin of the degraded device performance: in the course of device release, the silicon had been significantly roughened, which is expected to greatly increase light scattering. From the SEM image in Fig. 9 taken at the edge of the wet-etched opening, it is clear that silicon has also been consumed in those areas on the chip where it was not protected by the photoresist. The fact that the slot appears to have been eroded less than the outer surface of the device [compare Figs. 8(a) and 8(b)] suggests that diffusion has played a role in the attack on silicon during processing. While this is an especially severe example of damage, even more minor erosion of the silicon will be detrimental to device performance, as previously discussed in Sec. II.

**Fig. 10.**
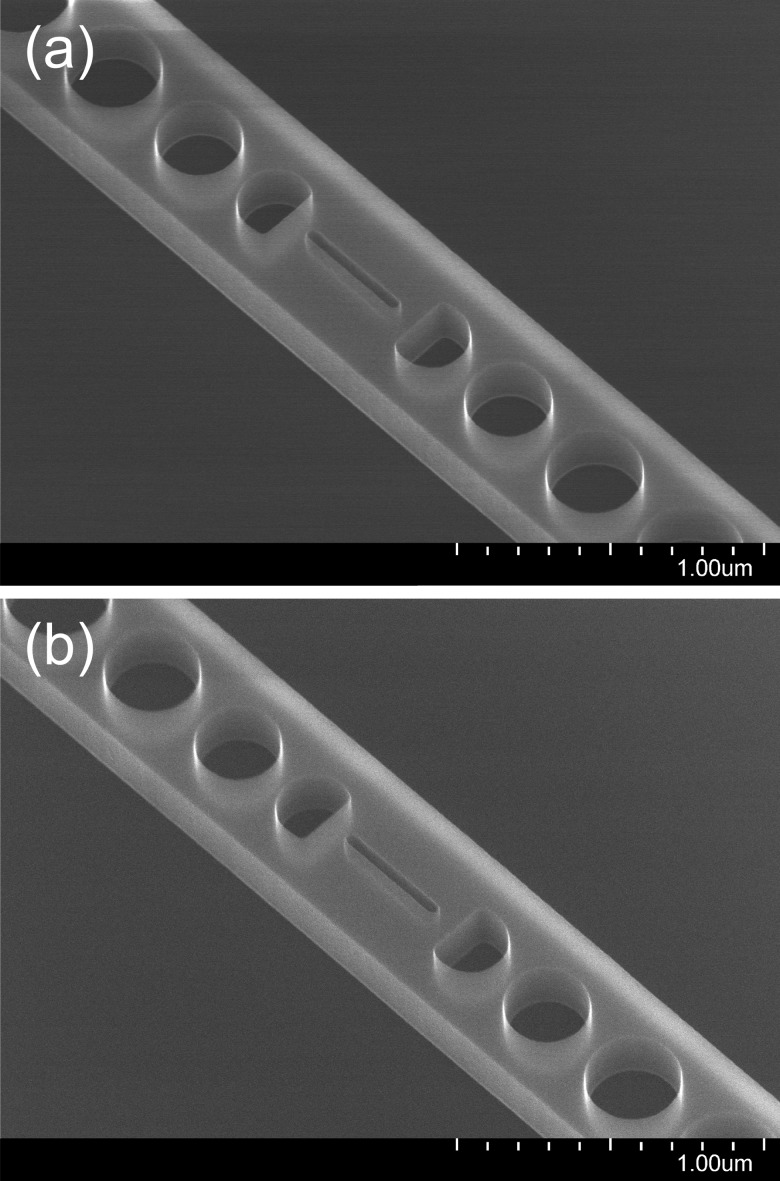
SEM images at 50 000× magnification and 30° tilt angle of the central slot region of the same slotted photonic crystal nanobeam cavity (a) before and (b) after exposure to diluted AZ 400 K developer for 30 s.

In principle, the silicon may have been attacked either during development of the photoresist or during wet etching with BOE. Development was performed by submerging and agitating the exposed chip for 30 s in a 1:4 mixture by volume of AZ 400 K (AZ Electronic Materials) to water. AZ 400 K is a buffered KOH solution. Since aqueous KOH can in fact be used to etch silicon, albeit usually at much higher concentration and elevated temperature (e.g., 80 °C),^23^ the effect of just the development step was tested on a SOI chip, on which the top silicon had been structured by ICP-RIE, but there was no photoresist. The before and after SEM images in Fig. 10 show no noticeable change in the silicon and indicate that the development step is innocuous.

**Fig. 11.**
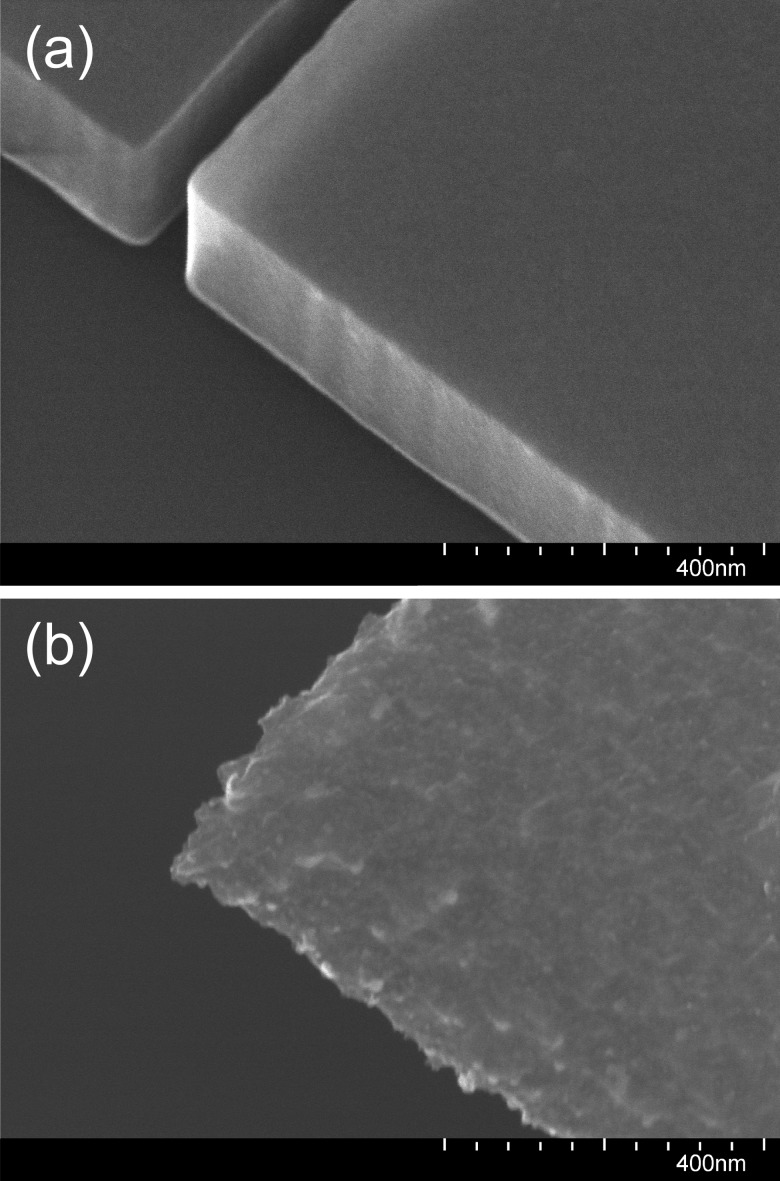
SEM images at 130 000× magnification and 30° tilt angle of a portion of a grating coupler at the same location (a) before and (b) after exposure to BOE for 15 min with continuous agitation. In image (b), a portion of the silicon structure has been lost due to undercutting during etching.

**Fig. 12.**
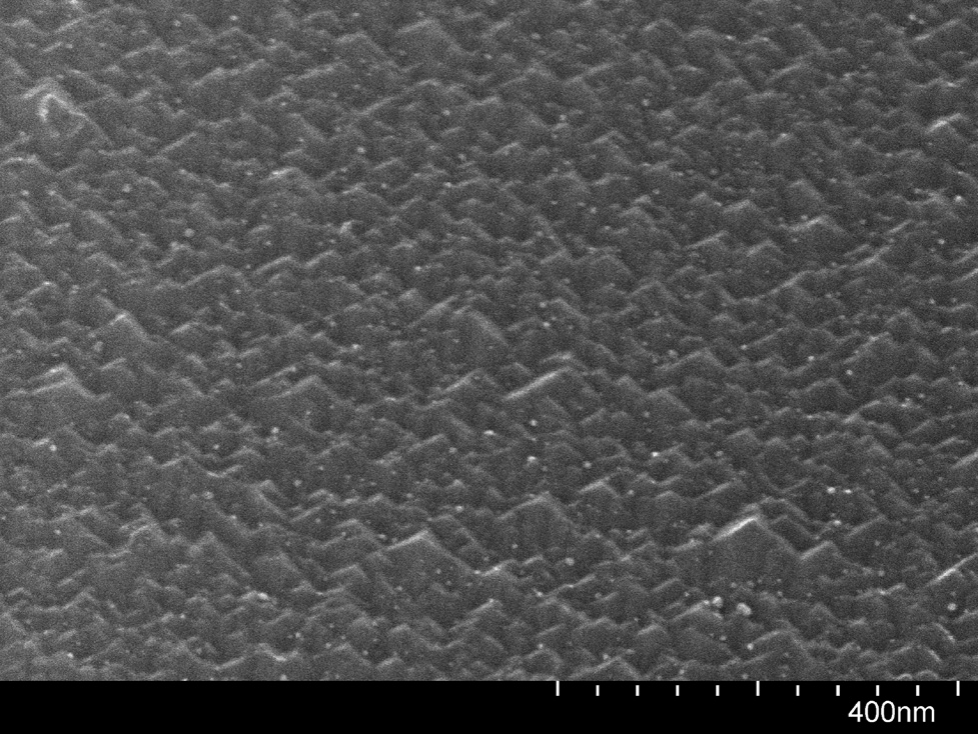
SEM image at 130 000× magnification and 30° tilt angle of the top surface of a grating coupler after exposure to BOE for 15 min with continuous agitation.

A similar test was made of the BOE wet etch step. Again, a chip with the top silicon patterned by ICP-RIE was employed without photoresist but this time without treatment with developer. The chip was submerged for 15 min in BOE solution a few millimeters deep, which was continuously swirled because there was already some indication that the damage to the devices was greater when the BOE solution was occasionally agitated. Figure 11 displays SEM images recorded before and after etching of the identical location (a portion of a grating coupler) on the chip. It can be seen that the silicon has been severely attacked. Examination of the top surface of the silicon reveals pyramidal structures (Fig. 12), which suggests that the roughening has some orientation-dependence, leaving what are presumably {111} planes exposed.

BOE is generally consider a highly selective etchant of SiO_2_ in the presence of silicon.^12–14^ However, attack by buffered HF on poly-silicon, leading to surface roughening, has been reported under various conditions, and has been correlated with the presence of contaminants, such as metals, as well as the deposition conditions or structure of the poly-silicon.^24–27^ Microroughening of single-crystal silicon during immersion in buffered HF solutions for very long times (1–5 days) has also been described.^28^ An infrared spectroscopy study of Si (111) surfaces exposed to buffered HF solutions of various *p*H values showed that the etch rate of these surfaces increased with OH^−^ concentration and suggested that oxidation of the surface Si-H species is an important and probably rate limiting step for etching.^29^ It is therefore likely that the device degradation observed here is caused by cyclic oxidation of silicon followed by etching of the resulting oxide. Indeed, etching of silicon with HNO_3_ and NH_4_F occurs via a mechanism of cyclic oxidation.^30^

In order to develop an improved process for device release, it is important to consider the nature of the etchant more closely.^12–14^ HF is a weak acid (pKa = 3.17) and does not completely dissociate into H_3_O^+^ and F^−^ ions in the usually employed aqueous solutions. In HF solutions buffered with NH_4_F, fluoride ions react with HF to form the hydrogen difluoride anion (HF_2_^−^), the concentration of which is typically greater than that of HF. HF_2_^−^ therefore becomes the dominate etchant species, as it attacks SiO_2_ about four to five times faster than HF.^31^ In concentrated aqueous HF, the even more highly reactive H_2_F_3_^−^ species appears to form. Use of NH_4_F as a buffer thus not only keeps the *p*H relatively constant, but stabilizes the etch rate as well by maintaining the concentrations of the various reactant species. Importantly, buffering with NH_4_F also ameliorates the problem of HF diffusion through photoresist causing loss of adhesion and thus loss of feature definition.^12,13^ Presumably, this is due to ionic species such as HF_2_^−^ and F^−^ being less soluble than HF in the photoresist.

Unbuffered aqueous solutions of HF in various concentrations were nevertheless tested as an alternative to BOE in the hope that a reduction in the concentration of the highly reactive HF_2_^−^ species would curtail the damage to silicon. In a test similar to those described above, where the sample was exposed to a 1:5 mixture by volume of concentrated HF (≥48%, Sigma Aldrich) to water for 15 min with continuous agitation to remove 0.73 *μ*m of SiO_2_, we find that attack of the silicon surface is markedly reduced, but not eliminated. Unfortunately, even 1:10 mixtures of concentrated HF to water eventually lead to loss of resist adhesion.

On the basis of the above considerations, we conclude that, in order to avoid attack and roughening of the silicon, the removal of SiO_2_ should be carried out at the fastest possible rate so as to outcompete diffusion of dissolved O_2_ to the surface of the silicon. Because of the depth of the etch and the inverse relation between etch-times and concentration, use of unbuffered HF solutions inevitably leads to photoresist delamination and is not viable. It is therefore difficult to completely avoid buffering with NH_4_F. A compromise is to use a mixture with a higher HF-to-NH_4_F ratio than standard BOE, to choose a photoresist with particularly good adhesion, to submerge the sample more deeply under the solution surface, and to avoid all agitation. Ideally, the etchant solution should be deoxygenated, for example by bubbling nitrogen or argon through the solution, but this is cumbersome and may not always be practical to implement.

The details of our optimized device release process, which removes about 1.1 *μ*m of SiO_2_, are presented in Table II. The initial cleaning with acetone and O_2_ plasma (carried out in a GIGAbatch 310 M from PVA TePla) are intended to remove any organic residues, for example from the Kapton tape used during dry etching. The AZ ECI 3000 (AZ Electronic Materials) family of photoresists are fast positive resists with high resolution capabilities, strong wet etch adhesion, and good thermal stability. Exposure with broadband ultraviolet light (365–415 nm) was performed with a MA6 mask aligner from Süss MicroTec. Following the postexposure bake, the resist is developed with AZ 726 MIF (AZ Electronic Materials), which as a metal-ion-free N(CH_3_)_4_OH solution should etch silicon even more slowly than a KOH-based developer, if at all. In fact, in an experiment similar to that carried out with AZ 400 K, we also observe no change in the silicon structure after exposure to undiluted AZ 726 MIF for 50 s with agitation. In any case, the development time was kept to a minimum. The hard bake after development improves adhesion, but can lead to embrittlement and cracking, and is therefore likewise kept to a short duration. It also makes the photoresist harder to remove, hence the use of *N*-methyl-2-pyrrolidone (NMP) to strip the resist after wet etching. Before wet-etching the sample, any residues of resist in the regions where the devices are to be released are removed by a mild O_2_ plasma treatment. The etch solution itself is a 1:5 mixture by volume of concentrated HF to BOE, for which there is still a slight molar excess of NH_4_F over HF. With this mixture, resist adhesion could be maintained well enough to produce a profile without significant undercutting at the edge of the device-release opening. It is however important to ensure that the concentrated HF and BOE are well mixed before immersing the chip, as a stratified mixture will expose the chip to a high concentration of HF and lead to delamination of the photoresist. The etching was carried out in a polytetrafluoroethylene vessel with enough etchant that the sample lay ca. 0.5–1 cm under the surface of the solution, and the solution was, of course, not agitated.

**Table II.** Optimized device release process.

Step	Description	Parameters
1	Cleaning	Acetone soak, ≥2 min
		Acetone rinse
		Isopropanol rinse
		N_2_ blow dry
2	O_2_ plasma clean	500 sccm O_2_, 600 W, 3 min, chip on glass
3	Dehydration	180 °C, 5 min
4	Hexamethyldisilazane treatment	110 °C
5	Spin coating of photoresist	AZ ECI 3027, 4000 rpm, 40 s, ramp 2000 rpm/s
6	Pre-exposure bake	90 °C, 90 s
7	Exposure	22.0 s, 13 mW/cm^2^ (290 mJ)
8	Postexposure bake	110 °C, 60 s
9	Development	AZ 726 MIF (undiluted), 50 s
		H_2_O rinse, ≥2 min
		N_2_ blow dry
10	Hard bake	180 °C, 5 min
11	O_2_ plasma treatment	500 sccm O_2_, 200 W, 1 min, chip on metal baseplate
12	Wet etch	1:5 by volume of HF (≥48%) to BOE, mix well, 3 min 15 s with no agitation
		H_2_O rinse, ≥2 min
		N_2_ blow dry
13	Resist strip	NMP soak on hotplate at 140 °C, ≥2 min
		Acetone rinse
		Isopropanol rinse
		N_2_ blow dry
14	O_2_ plasma clean	500 sccm O_2_, 600 W, 3 min, chip on glass

**Fig. 13.**
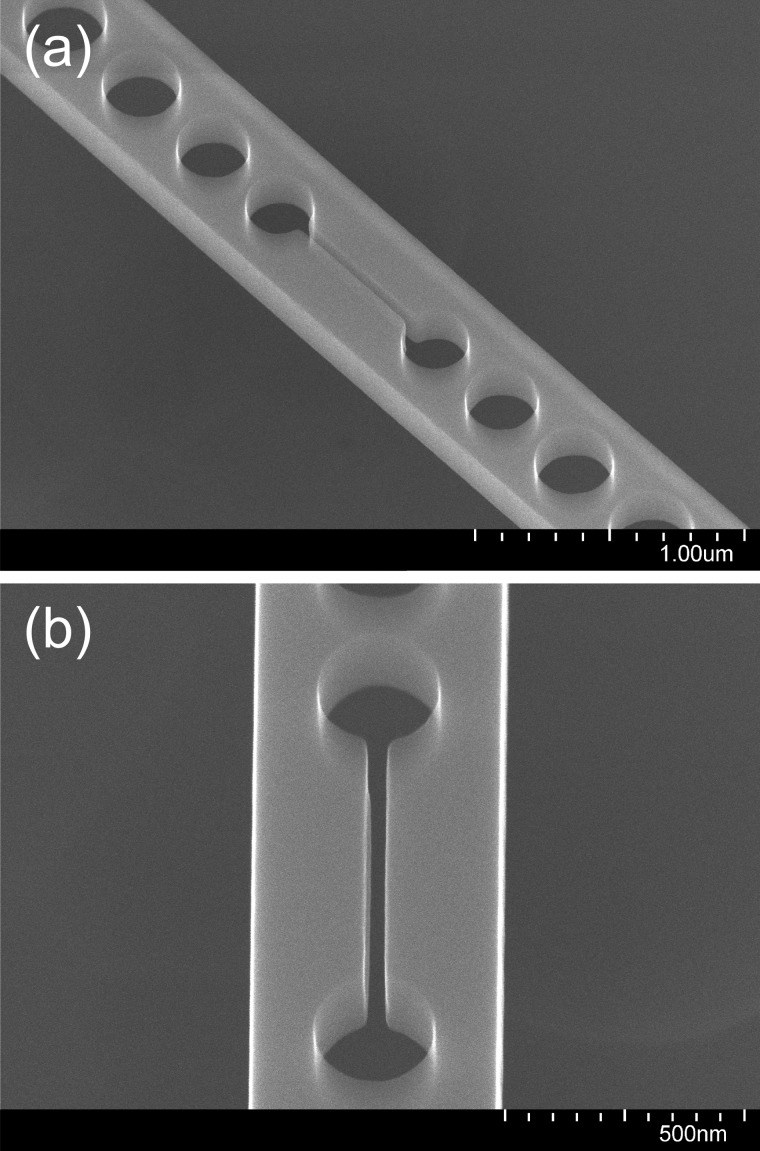
SEM images of the central slot region of the same fully processed, free-standing, slotted photonic crystal nanobeam cavity viewed at two different angles: (a) at 45 000× magnification and 30° tilt angle and (b) 80 000× magnification and 30° tilt angle.

**Fig. 14.**
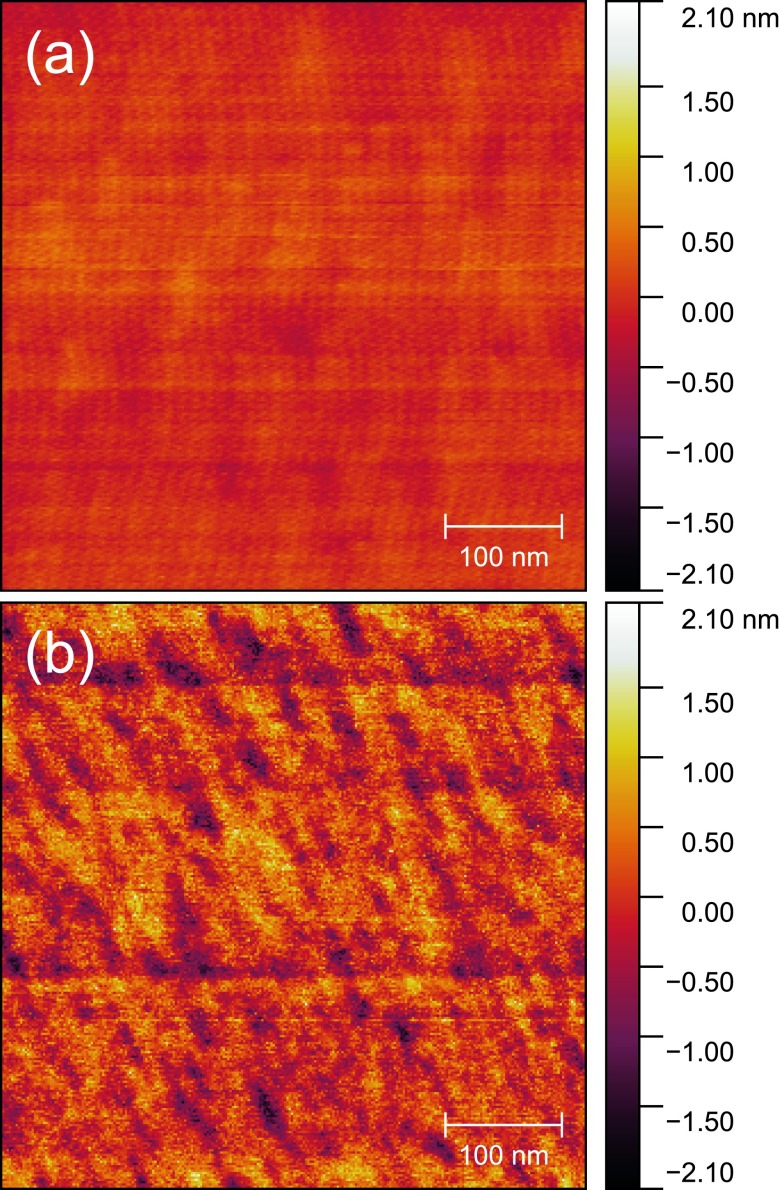
(Color) AFM images of a silicon surface (a) before and (b) after exposure to a 1:5 mixture by volume of concentrated HF to BOE for 3 min 15 s with no agitation.

The high quality of the devices produced using the optimized dry-etch and wet-etch procedures described above is evident from the SEM images of a free-standing device displayed in Fig. 13. To confirm that the silicon surface is in fact not significantly degraded by step 12 in Table II, this step was carried out with a test chip on which the top silicon had been structured by ICP-RIE, but there was no photoresist, thus leaving relatively large areas of silicon unprotected that could be inspected with an atomic force microscope (AFM). As can be seen from the AFM images shown in Fig. 14 of the top surface of the silicon before and after exposure to the wet etching solution, the roughness increases only slightly, in this case from a root-mean-square value of 0.146 nm to a value of 0.473 nm.

**Fig. 15.**
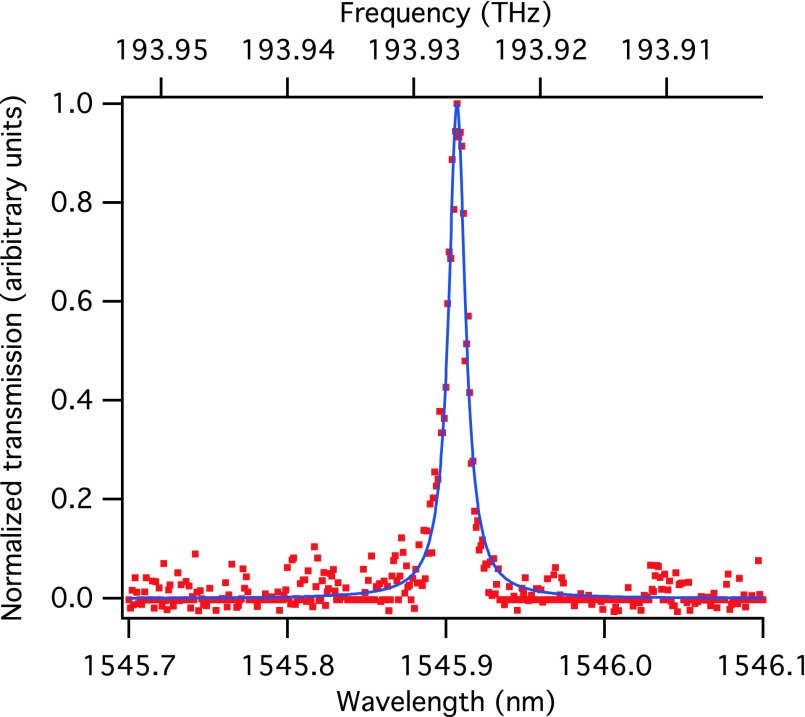
(Color) Normalized transmission spectrum (red dots) of a slotted photonic crystal nanobeam cavity fabricated with the optimized dry-etch and wet-etch processes. The blue line is a fit of a Lorentzian function to the data, for which the full width at half maximum is 1.59 GHz, corresponding to a Q of 1.22 × 10^5^. The simulated wavelength and Q are 1554 nm and 1.2 × 10^6^, respectively.

Even more telling are the optical transmission measurements; it is found that the optical resonance frequency of the photonic crystal cavity is reproducible within 1% of the simulated value and quality factors on the order of 10^5^ are routinely achieved. An example is shown in Fig. 15.

## IV. SUMMARY AND CONCLUSIONS

We have presented an optimized method for fabricating free-standing photonic devices in silicon that ensures both dimensional integrity and smooth, nearly vertical side walls. A means for avoiding undercutting during dry etching of small, high-aspect ratio openings by making an electrically conducting connection to the top surface of the sample has been described. Although demonstrated here for SOI substrates, preliminary results suggest that this procedure may also work for other materials, such as compound semiconductors. The problem of silicon loss and surface roughening during device release with HF chemistry was addressed with an approach aimed at minimizing exposure to atmospheric oxygen. In the future, we hope to develop a practical procedure for wet etching in an inert atmosphere, which should allow the use of a greater range of etchant solutions. A quasi-one-dimensional photonic crystal cavity served as an example for elucidating typical geometric tolerances of such devices. Importantly, in the experimental methods described here, no iterative process to adjust as-processed dimensions to design dimensions has been employed.
